# The influence of the pituitary tumor transforming gene-1 (PTTG-1) on survival of patients with small cell lung cancer and non-small cell lung cancer

**DOI:** 10.1186/1477-3163-5-4

**Published:** 2006-01-20

**Authors:** Nina Rehfeld, Helene Geddert, Abedelsalam Atamna, Astrid Rohrbeck, Guillermo Garcia, Slawek Kliszewski, Judith Neukirchen, Ingmar Bruns, Ulrich Steidl, Roland Fenk, Helmut E Gabbert, Ralf Kronenwett, Rainer Haas, Ulrich-Peter Rohr

**Affiliations:** 1 Klinik für Hämatologie, Onkologie und klinische Immunologie, Heinrich-Heine-Universität Düsseldorf, Moorenstraße 5, D-40225 Düsseldorf, Germany; 2 Institut für Pathologie, Heinrich-Heine-Universität Düsseldorf, Moorenstraße 5, D-40225 Düsseldorf, Germany

## Abstract

**Background:**

PTTG-1 (pituitary tumor transforming gene) is a novel oncogene that is overexpressed in tumors, such as pituitary adenoma, breast and gastrointestinal cancers as well as in leukemia. In this study, we examined the role of PTTG-1 expression in lung cancer with regard to histological subtype, the correlation of PTTG-1 to clinical parameters and relation on patients' survival.

**Methods:**

Expression of PTTG-1 was examined immunohistochemically on formalin-fixed, paraffin-embedded tissue sections of 136 patients with small cell lung cancer (SCLC) and 91 patients with non-small cell lung cancer (NSCLC), retrospectively. The intensity of PTTG-1 expression as well as the proportion of PTTG-1 positive cells within a tumor was used for univariate and multivariate analysis.

**Results:**

PTTG-1 expression was observed in 64% of SCLC tumors and in 97.8% of NSCLC tumors. In patients with SCLC, negative or low PTTG-1 expression was associated with a shorter mean survival time compared with patients with strong PTTG-1 expression (265 ± 18 days vs. 379 ± 66 days; p = 0.0291). Using the Cox regression model for multivariate analysis, PTTG-1 expression was a significant predictor for survival next to performance status, tumor stage, LDH and hemoglobin.

In contrast, in patients with NSCLC an inverse correlation between survival and PTTG-1 expression was seen. Strong PTTG-1 expression was associated with a shorter mean survival of 306 ± 58 days compared with 463 ± 55 days for those patients with no or low PTTG-1 intensities (p = 0.0386). Further, PTTG-1 expression was associated with a more aggressive NSCLC phenotype with an advanced pathological stage, extensive lymph node metastases, distant metastases and increased LDH level. Multivariate analysis using Cox regression confirmed the prognostic relevance of PTTG-1 expression next to performance status and tumor stage in patients with NSCLC.

**Conclusion:**

Lung cancers belong to the group of tumors expressing PTTG-1. Dependent on the histological subtype of lung cancer, PTTG-1 expression was associated with a better outcome in patients with SCLC and a rather unfavourable outcome for patients with NSCLCs. These results may reflect the varying role of PTTG-1 in the pathophysiology of the different histological subtypes of lung cancer.

## Introduction

Progress of cancer research on molecular level led to the discovery and a better understanding of genes involved in the development of tumor growth. In 1997, a new proto-oncogene was identified in pituitary growth hormone secreting tumor cells leading to a more malignant phenotype [1]. As a result, it was termed pituitary tumor-transformation gene (PTTG-1). The 23 kDa PTTG-1 protein consists of 202 amino acids and is located on chromosome 5q33, a locus previously associated with progression of lung cancer [2-4]. PTTG-1 is physiologically expressed in normal tissues of different organs such as testis, colon and lung while an overexpression was observed in several malignant tumors and cancer cell lines, including carcinomas of the gastrointestinal tract, breast, lung, as well as in leukemia and lymphoma [5-8]. The possible role of PTTG-1 as a proto-oncogene *in vivo *was exemplified when NIH 3T3 fibroblasts were transfected with the full-length coding sequence of PTTG-1. The transfection of PTTG-1 lead to morphological changes, alterations in growth characteristics of the fibroblasts and tumor formation resulted when injected into nude mice. Irrespective of the compelling evidence that PTTG-1 is a novel oncogene, the mechanisms underlying the induction of malignant cell transformation are not understood. Some reports suggest that PTTG-1 plays a role in the regulation of transcription [9,10] such as induction of basic fibroblast growth factor [11] or activation of c-Myc [12] leading to increased angiogenesis as well as cell proliferation. PTTG-1 might also be related to cell cycle genes and the induction of apoptosis depending on the particular type of cell [12-14].

In the present study, we examined the prognostic value of PTTG-1 expression for survival in relationship to the different histological subtypes of lung cancer. The intensity of PTTG-1 staining as well as the proportion of PTTG-1 positive cells within a tumor was taken into consideration for univariate and multivariate survival analyses.

## Patients and methods

### Characteristics of the patients with SCLC and NSCLC

Formalin-fixed, paraffin-embedded tissue sections from 136 patients with primary SCLC and 91 patients with primary NSCLC were used for immunohistochemical detection of PTTG-1. Specimens were obtained from the Institute of Pathology of the University of Duesseldorf. Biopsies from primary lung tumor were taken at initial clinical presentation. The original diagnosis of SCLC or NSCLC was confirmed by two different pathologists before the biopsy was accepted for this study. The histopathological diagnosis was based on HE stains in each cases and confirmed by immunohistochemical staining of cytokeratin 5 and 7, chromogranin A, synaptophysin and TTF-1. 91 NSCLC tumors were classified into histological subtypes: 55 adenocarcinomas (60.4%), 28 squamous cell carcinomas (30.8%), six large cell carcinomas (6.6%) and two adeno-squamous cell carcinomas (2.2%). The patients enrolled in the study were patients from the University of Duesseldorf and associated academic hospitals of the University of Duesseldorf between 1983 and 2003. Clinical data of the patients were collected from chart review with given approval from the ethics committee of the University of Duesseldorf. Baseline characteristics of chosen patients are presented in Table 1 and 2. Survival time in days was calculated from the date of histopathological diagnosis. Performance status of the patients was evaluated by applying standard WHO criteria. All tumors were classified according the current TNM system [15]. The stages I- IV were based on the revised version of the International System for Staging Lung Cancer [16]. Chemotherapy was perfomed as a first-line treatment with an average of 3.5 cycles (median: 3 ± 1.9 cycles; range: 1–12) and 3.1 cyles (median: 3 ± 2.1 cycles; range: 1–11) in all SCLC patients and 93.4% of NSCLC patients, respectively. After recurrence of the tumor following surgical resection, the remaining 6.6% patients with NSLCL were treated with palliative chemotherapy.

**Table 1:** Baseline characteristics of patients with SCLC (N = 136)

	Patients (%)
Mean age (years)	60.8 ± 9.5 (range: 42.2–82.2)
Sex	
Male	103 (75.7%)
Female	33 (24.3%)
Smoking status	
Smoker/former smoker	120 (88.2%)
Nonsmoker	6 (4.4%)
Not evaluable	10 (7.4%)
WHO performance status	
0	48 (35.3%)
I	61 (44.9%)
II	18 (13.2%)
III	2 (1.5%)
Not evaluable	7 (5.1%)
Stage	
Ia,b	1 (0.7%)
IIa,b	7 (5.1%)
IIIa,b	43 (31.6%)
IV	82 (60.3%)
Not evaluable	3 (2.2%)
LDH (units/L)	333.2 ± 233.3
Hemoglobin (g/dl)	13.4 ± 1.7
Thrombocytes (μl)	322416.7 ± 114639.9
Leukocytes (μl)	9208.9 ± 3017.9

**Table 2:** Baseline characteristics of patients with NSCLC (N = 91)

	Patients (%)
Mean age (years)	62.6 ± 10.9 (range:31.5–87.8)
Sex	
Male	67 (73.6%)
Female	24 (26.4%)
Smoking status	
Smoker/former smoker	84 (92.3%)
Nonsmoker	5 (5.5%)
Not evaluable	2 (2.2%)
WHO performance status	
0	34 (37.4%)
I	45 (49.4%)
II	10 (11.0%)
Not evaluable	2 (2.2%)
Stage	
Ia,b	3 (3.3%)
IIa,b	4 (4.4%)
IIIa,b	27 (29.7%)
IV	57 (62.6%)
LDH (units/L)	229.7 ± 142.1
Hemoglobin (g/dl)	13.4 ± 1.7
Thrombocytes (μl)	320736.3 ± 178681.1
Leukocytes (μl)	10319.8 ± 4925.3

The preferred chemotherapeutic regiment for 71.5% of SCLC patients was a combination of cyclophosphamid (1000 mg/m^2 ^d1), epirubicin (65 mg/m^2 ^d1) or adriamycin (45 mg/m^2^) and etoposid (120 mg/m^2^). A platinum-based combination with cisplatin (90 mg/m^2^) or carboplatin (300 mg/m^2^) and etoposid (150 mg/m^2^) was given in 18%, whereas other combinations were given in 10.5% of patients.

For 70.3% of NSCLC patients the preferred chemotherapeutic regiment was a platinum based combination (cisplatin 75 mg/m^2 ^or carboplatin AUC 5). In detail, 26.4% of patients received a combination of platin and etoposid (100 mg/m^2^); 19.7% of patients had a therapy based on platin and paclitaxel (175 mg/m^2^) and 14.3% of patients were treated with platin and vinorelbine (30 mg/m^2^). Finally, 9.9% of patients had a cisplatin monotherapy supported by radiotherapy. 12.1% of patients received a combination of gemcitabine (1000 mg/m^2^) and vinorelbine (30 mg/m^2^). Other chemotherapeutic combinations were given in 17.6% of cases.

### Immunohistochemistry

As previously described by Saez et al. [17], a standardized immunohistochemical staining procedure was adhered to PTTG-1 detection, using a primary polyclonal antibody against PTTG-1 (U.S. Biological, Massachusetts, USA). Briefly, fresh tumor tissue specimens were immediately formalin-fixed following bronchoscopy or computertomographic-supported thoracal puncture. Samples were paraffin-embedded, cut in 2- to 4 μm sections on poly-L-lysine-coated slides. Following deparaffinization with xylene (15 minutes), sections were rehydrated through decreasing concentrations of ethanol (100%, 96% and 70%) and washed for 5 minutes. For antigen retrieval, the specimens were heated in citrate buffer (pH 6) using a pressure cooker for 15 minutes. Endogenous peroxidase activity was eliminated by incubation with 3% hydrogen peroxide for 15 minutes and unspecific binding of biotin and avidin was blocked using a blocking solution (Dako, Hamburg, Germany) for 15 minutes each slide. After intensive washing for 5 minutes, slides were incubated with 1% bovine serum albumin (Sigma, St.Louis, MO) to block unspecific binding of the primary antibody.

The anti-PTTG-1 antibody (U.S. Biological, Massachusetts, USA) was diluted 1:100, and slides were incubated with anti-PTTG-1 antibody for 1 hour. After washing, a streptavidin horseradish peroxidase detection kit (DAKO, Hamburg, Germany) containing 3,3'-diaminobenzidine solution as substrate was used for immunohistochemical staining according to the manufacturer's instructions. Appropriate positive controls (testis and colon tumor tissue) and appropriate negative control (kidney) were additionally evaluated in each run. All slides were simultaneously assessed by two investigators (N.R. and A.A.) using a double-headed discussion microscope. In a final control process all slides were reviewed and the results were confirmed by a third investigator (H.G.). The staining intensities for PTTG-1 were evaluated semiquantitatively and classified into three groups. The first group showed equal or stronger cytoplasmic staining intensity compared with positive control. The second group showed lower staining intensity of PTTG-1 compared with the positive control. The third group showed no immunohistochemical evidence of PTTG-1 expression. For the estimation, all tumor areas of one to four biopsies from bronchoscopy were included. Generally, the staining intensity and the proportion of PTTG-1 positive cells were uniform within the biopsies. For those few cases with a difference in staining intensity, the strongest intensity was chosen for the assessment of PTTG-1. The proportion of positive cells was determined semiquantitatively: (I) none, (II) between 1% and 50%, (III) between 51% and 75%, and (IV) more than 75% of the tumor cells with PTTG-1 expression. The percentage of positive cells was estimated in agreement with all three observers.

### Statistical methods

Immunohistochemical staining was performed without prior knowledge of clinical parameters. Mean and standard error are reported for several clinical parameters. The Kaplan-Meier survival analysis with log rank test was used to compare clinical parameters with PTTG-1 expression. Two sided *t*-test was performed to compare clinical variables of PTTG-1 positive, PTTG-1 low positive and PTTG-1 negative group. For multivariate analysis, a Cox regression model with a forward stepwise selection was used. The following variables were included for multivariate analysis: gender (male versus female), age (≤ 60 versus > 60 years), performance status (classified into WHO 0 vs. I vs. II vs. III), tumor stage (WHO classification), metastasis (no metastasis vs. metastasis), hemoglobin level (< 12 versus ≥ 12 mg/dl), platelet count (< 150.000/μl, 150.000–400.000 μl, > 400.000 μl), LDH level (classified into serum levels ≤ 240 versus > 240 units/L), leucocyte count (≤ 11.000/μl versus >11.000/μl) as well as staining intensitiy of PTTG-1 expression (PTTG-1 negative/ low versus PTTG-1 strong) and quantitative PTTG-1 expression (PTTG-1 expressing tumor cell proportion of ≤ 50% versus > 50%). Pearson's bivariant correlation was performed to evaluate a correlation between PTTG-1 expression or proportion of PTTG-1-expressing cells to clinical parameters. Statistical analysis was performed using SPSS software 12.0. Significance is defined as p < 0.05 and the respective values are given in the text.

## Results

### Immunostaining of PTTG-1 in tumors from patients with SCLC

**Figure 1:**
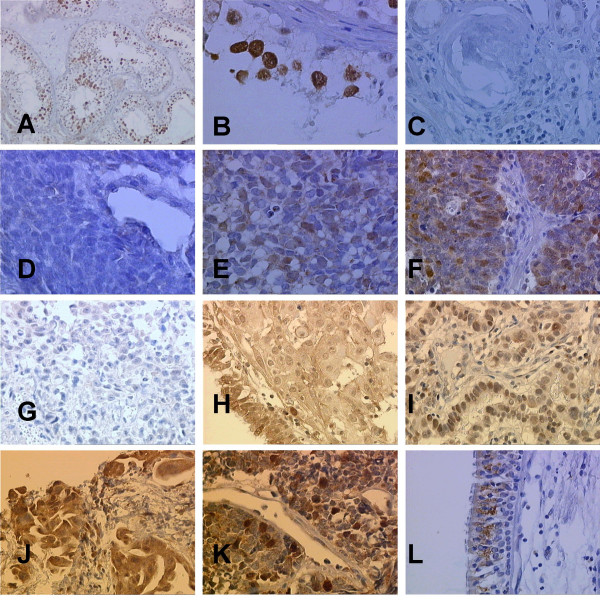
*Immunohistochemical staining of PTTG-1*. **(A + B) **positive control of germ cells in testis; **(C) **negative control of uropoietic tubuli stroma cells and globuli of the kidney; SCLC with negative PTTG-1 expression **(D)**; low PTTG-1 expression **(E) **and strong PTTG-1 expression **(F)**. Adenocarcinoma (NSCLC) with negative PTTG-1 expression **(G)**, low PTTG-1 expression **(H) **and strong PTTG-1 expression **(I)**. Squamous cell carcinoma (NSCLC) with low PTTG-1 expression **(J) **and strong PTTG-1 expression **(K)**. **(L)**, note the positive cytoplasmatic staining of normal bronchial epithelium.

PTTG-1 expression was observed in 87 of 136 examined tumors (64%). With regard to the degree of expression low cytoplasmatic staining intensity was found in 60 cases (44.1%) and strong cytoplasmatic staining intensity was found in 27 cases (19.9%), respectively. There was no PTTG-1 expression in 49 (36%) cases (Figure 1).

**Figure 2:**
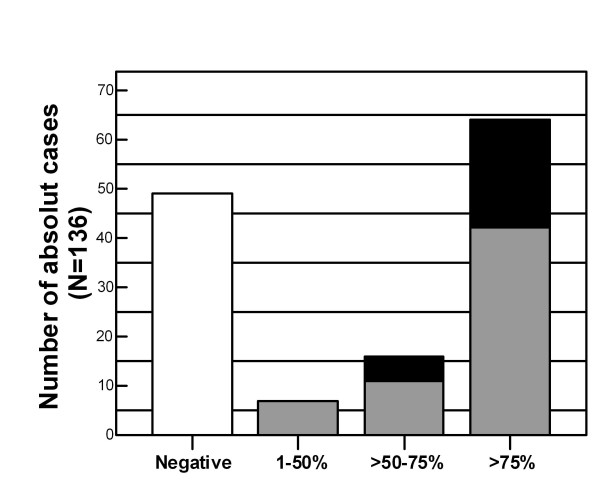
Proportion of PTTG-1 positive tumor cells and PTTG-1 staining intensities of SCLC (N = 136); *white bar *represents PTTG-1 negative cases; *light grey *and *black bars *represent PTTG-1 low and strongly stained SCLC tumors, respectively.

Positive tumors mostly showed more than 75% of stained cells (64/136; 47.1%). Proportion of PTTG-1 positive cells between 1% and 50%, and between 51% and 75% were found in 7 (5.1%) and 16 (11.8%) of all tumors, respectively, whereas 49 (36%) were PTTG-1 negative (Figure 2).

### Prognostic value of PTTG-1 in patients with SCLC using Kaplan-Meier survival curves

**Figure 3:**
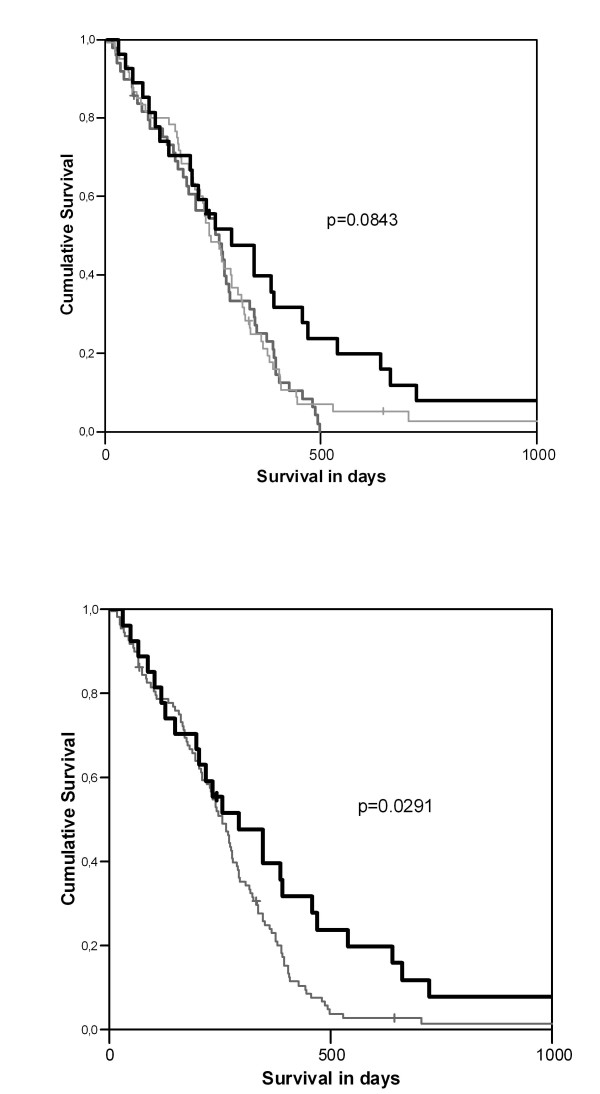
**3A and 3B ***Association of PTTG-1 expression and survival in SCLC tumors*. (A): no statistical differences in survival time between patients with SCLC of no, low or strong PTTG-1 expression were seen (p = 0.0843). (B): a statistical significant difference on survival time was observed (p = 0.0291), when comparing those patients with PTTG-1 negative/low expression (grey line) with those patients with strong PTTG-1 expression (black line). PTTG-1 strong expressing cases (black line) had a statistical longer mean survival time of 379 ± 66 days compared with 265 ± 18 days for those patients with no/low PTTG-1 expression (grey line).

Median and mean survival rates for all 136 patients with SCLC were 255 ± 16 days and 287 ± 20 days. One year and five year survival rates were 26.9% and 0%, respectively. To evaluate the relationship of different PTTG-1 intensities on survival, the survival curves of the three groups showing no, low or strong PTTG-1 staining intensities were compared. Differential expression of PTTG-1 was not associated with survival up to 300 days (p = 0.0843; Figure 3A). Of notice, beyond 300 days, a significant improved survival rate was observed for those patients with strong PTTG-1 expression compared to those with no or low PTTG-1 expression. No significant difference was observed between the survival curves of patients with no or low PTTG-1 staining (mean survival: 249 ± 20 days versus 278 ± 29 days, p = 0.7026). Therefore, PTTG-1 negative or low stained cases were grouped together for further analysis.

Patients with negative or low expression of PTTG-1 had a significant (p = 0.0291) shorter mean survival of 265 ± 18 days compared with 379 ± 66 days for those patients with strong PTTG-1 expression (Figure 3B). No significant difference was observed between the two groups (PTTG-1-negative/low vs. PTTG-1 strong) with respect to gender, age, smoking status, performance status, tumor stage, mean cycles of chemotherapy, radiotherapy, mean hemoglobin level, mean platelet count, mean leukocyte count and mean lactate dehydrogenase using two sided t-test.

Next, the proportion of PTTG-1 expression was correlated to survival of the SCLC patients. In order to evaluate the proportion of PTTG-1 expression in SCLC: tumors with ≤ 50% PTTG-1 expression (including negative cases) and tumors with > 50% of PTTG-1 expression were compared. A trend to significance in mean survival of 241 ± 18 days (PTTG-1 expression ≤ 50%) versus 319 ± 31 days (PTTG-1 expression > 50%) was observed for the two groups (p = 0.0541). Both groups did not show statistical differences with respect to other clinical parameters.

### Clinical parameters and PTTG-1 expression in multivariate analysis for patients with SCLC

Next to PTTG-1 status, other clinical parameters such as performance status (WHO 0/I vs. II/III; p= 0.0007), tumor stage (I/II vs. III/IV; p = 0.0117) and LDH level (serum levels ≤ 240 units/l vs. > 240 units/l; p = 0.0246) were also related to survival time using Kaplan-Meier analysis. According to Pearson's bivariant correlation analysis PTTG-1 expression was not correlated to any other clinical variable. In order to test PTTG-1 as an independent prognostic factor, multivariate anaysis was performed. As a result, the staining intensity of PTTG-1 expression (p = 0.047), tumor stage (p = 0,051), performance status (p = 0.015), LDH level (p = 0.001) and hemoglobin level (p= 0.011) were identified as independent prognostic parameters by the Cox regression modell (Table 3). In contrast, the percentage of PTTG-1 positive tumor cells, thrombocyte count, leucocyte count, gender and age were irrelevant with regard to survival time (p > 0.2).

**Table 3:** Variables accepted in the forward selection model of the Cox regression as explanatory factors in SCLC patients

	p-value
Staining intensity of tumor cells expressing PTTG-1^a^	0.047
Stage^b^	0.051
Performance status^c^	0.015
LDH^d^	0.001
Hemoglobin^e^	0.011

^a^-negative/low PTTG-1 expressing tumor cells vs. PTTG-1 strong expressing tumor cells;

^b^-according to TNM-system: stage I/II vs. III/IV;

^c^-performance status according to WHO 0 vs. I vs. II vs. III;

^d^-LDH serum levels ≤ 240 units/l vs. > 240 units/l;

^e^-hemoglobin levels ≤ 12 g/dl vs. >12 g/dl

### Immunostaining of PTTG-1 in tumors from patients with NSCLC

PTTG-1 expression was observed in 89 of 91 examined tumors (97.8%). With regard to the degree of PTTG-1 expression low PTTG-1 staining intensity was found in 52 cases (57.1%) and strong cytoplasmatic PTTG-1 staining intensity was found in 37 cases (40.7%). There was no PTTG-1 expression in 2 (2.2%) cases (Figure 1).

**Figure 4:**
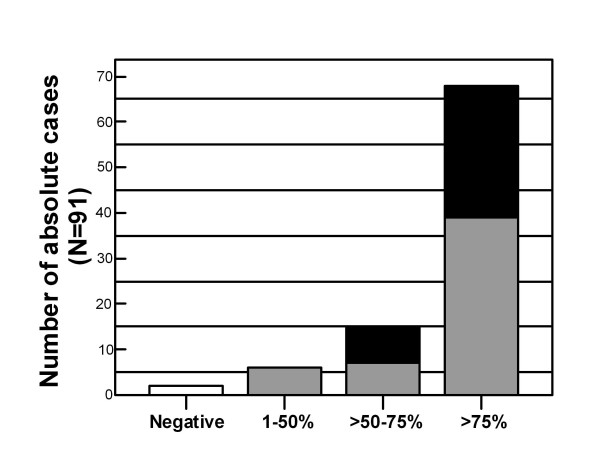
Proportion of PTTG-1 positive tumor cells and PTTG-1 staining intensities of NSCLC (N = 91); *white bar *represents PTTG-1 negative cases; *light grey *and *black bars *represent PTTG-1 low and strongly stained NSCLC tumors, respectively.

Positive tumors mostly showed more than 75% of stained cells (68/91; 74.7%). Proportions of PTTG-1 positive tumor cells between 1% and 50%, between 51% and 75% were found in 6 (6.6%) and 15 (16.5%) of all tumors, respectively, only 2 (2.2%) cases were PTTG-1 negative (Figure 4).

### Prognostic value of PTTG-1 in patients with NSCLC using Kaplan-Meier survival curves

Median and mean survival rates for all 91 patients with NSCLC were found to be 401 ± 41 days and 325 ± 56 days. Patients with NSCLC had one year and five year survival rates of 46.6% and 1.2%, respectively.

**Figure 5:**
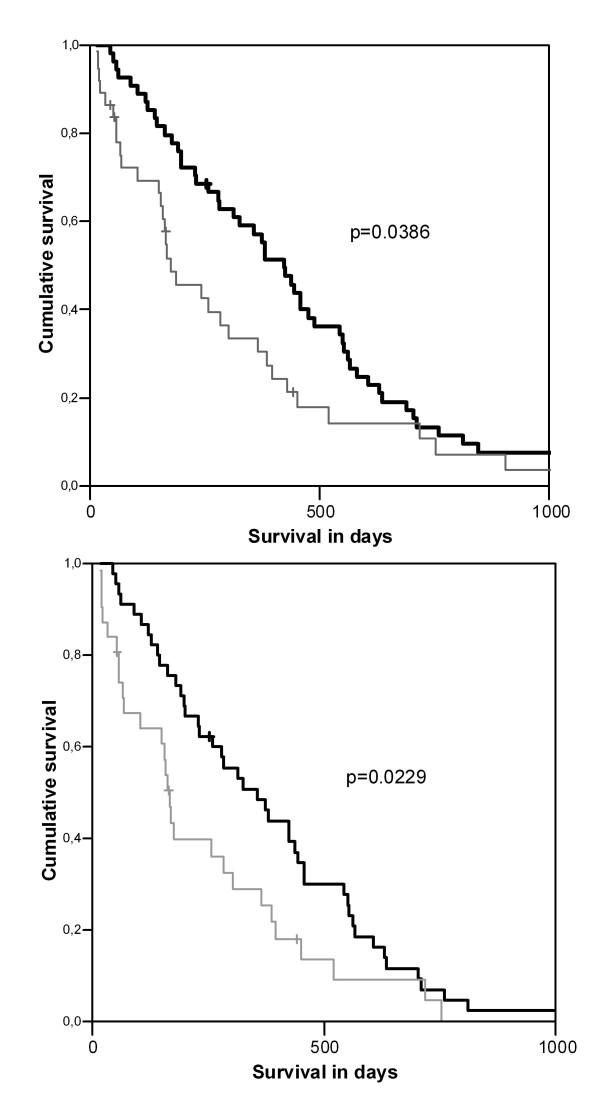
**5A and 5B ***Association of PTTG-1 expression and survival in NSCLC tumors*. (A): statistical difference in survival time between patients with NSCLC (n = 91) of no/low PTTG-1 expression (black line) and strong PTTG-1 expression (grey line) was seen. Patients with strong PTTG-1 expression had a significant (p = 0.0386) shorter survival time. (B): looking at PTTG-1 expression for the subgroup of patients with advanced NSCLC receiving palliative chemotherapy (n = 76), a statistically significant difference on survival time between PTTG-1 no/low expressing group (black line) and PTTG-1 strong expressing group (grey line) was observed (p = 0.0229).

No significant difference between median and mean survival was observed for tumors from patients with no or low PTTG-1 expression (p = 0.3063). Consequently, negative and low stained cases were grouped together. As a result PTTG-1 expression was negatively correlated with survival. Patients with strong PTTG-1 expression had an unfavourable prognosis showing a mean survival of 306 ± 58 days compared with patients with PTTG-1 low or PTTG-1 negative stainings with a mean survival of 463 ± 55 days (p = 0.0386; Figure 5A).

In order to exclude a possible bias due to different therapies, surgically resected patients, who had a significant better overall survival than unresected patients (p = 0.0001), were removed from the analysis. Looking at the subgroup of patients in stage IIIB and IV (n = 76) receiving palliative chemotherapy, a significant difference on survival (p = 0.0229) with regard to PTTG-1 expression was observed again (Figure 5B).

In order to evaluate a possible correlation of clinical variables to PTTG-1 expression, the two sided t-test was used. When comparing patients group with strong PTTG-1 expression versus negative/low PTTG-1 expression statistically significant differences were noticed with respect to gender (p = 0.04), LDH level (p = 0.005), leucocyte count (p = 0.016) and tumor stage (p = 0.021). Using Pearson's bivariant correlation analysis (p < 0.05) a strong expression of PTTG-1 was associated with advanced LDH level, lymphnode involvement and distant metastases. In contrast, no correlation with PTTG-1 expression was found for the following clinical variables: age, smoking status, performance status, mean cycles of chemotherapy, radiotherapy, erythropoetin substitution, mean hemoglobin level and mean platelet count.

### Clinical parameters and PTTG-1 expression in multivariate analysis in patients with NSCLC

Among the clinical parameters, performance status (WHO 0 vs. I vs. II), LDH (serum levels ≤ 240 units/l vs. > 240 units/l), leucocyte count (< 11.000/nl vs. ≥ 11.000/nl) and TNM classification (stage I vs. II vs. II vs. IV) were also related to survival time using Kaplan-Meier analysis for NSCLC (data not shown). According to Pearson's bivariant correlation analysis PTTG-1 expression was correlated to increased LDH level (p < 0.01) and leucocyte count (p < 0.05), extended lymph node involvement (p < 0.05) and distant metastases (p < 0.01). To examine the influence of PTTG-1 expression as an independent prognostic factor for unresectable NSCLC patients multivariate analysis was performed. As a result, only PTTG-1 staining intensity (p = 0.025), tumor stage (p = 0.044) and performance status (p = 0.016) were identified as independent prognostic parameters by the Cox regression model (Table 4). In contrast, gender, age, hemoglobin level, thrombocyte count, leucocyte count, LDH level and proportion of PTTG-1 expression were irrelevant with regard to survival time (p > 0.05).

**Table 4:** Variables accepted in the forward selection model of the Cox regression as explanatory factors in NSCLC patients

	p-value
Staining intensity of tumor cells expressing PTTG-1^a^	0.025
Stage^b^	0.044
Performance status^c^	0.016

^a^-negative/low PTTG-1 expressing tumor cells vs. PTTG-1 strong expressing tumor cells;

^b^-according to TNM-system: stage I-III vs. IV;

^c^-performance status according to WHO 0 vs. I vs. II

## Discussion

In this study a high prevalence of PTTG-1 expression in lung cancer was seen. We showed that 64% of SCLC tumors and 97.8% of NSCLC tumors examined by immunohistochemistry were positive for PTTG-1 expression. To our knowledge, only a few reports refer to the presence of PTTG-1 in lung cancer. In line with our results a PTTG-1 expression was observed in 9 out of 10 primary adenocarcinomas of the lung using immunohistochemial methods [17]. Performing RT-PCR for the detection of PTTG-1 mRNA Honda et al. detected PTTG-1 expression in 12 out of 14 NSCLCs. Interestingly, squamous cell carcinomas had a tendency to express more PTTG mRNA than adenocarcinomas [18].

Next, the prognostic relevance of PTTG-1 expression on survival of the patients was examined. A strong PTTG-1 expression was significantly associated with favourable prognosis for patients with SCLC prolonging the mean survival for 3.5 months. In contrast, patients with NSCLC showing strong PTTG-1 tumor expression had a rather unfavourable overall survival which was 5.2 months lower than the group of patients with negative or low PTTG-1 expression. At first glance our results seem to be contradictory but might be a reflection of the varying role of PTTG-1 in the pathophysiology of the different histological subtypes of lung cancer. This view is not entirely hypothetical when looking at the literature. On one hand, recent studies indicate that overexpression of PTTG-1 was associated to induce p53 independent and p53-dependent apoptosis [13,19] when looking at breast cancer cells or kidney cell lines. Further, PTTG-1 overexpression in the human lung cancer cell line A549 inhibited the cell growth via activation of p21 [20]. In contrast to these results, others suggest that PTTG-1 expression led to an increased rate of cell growth or inhibition of apoptosis. For example, p53 mediated cell death of NSCLC lung cancer cell line H1299 was impeded by PTTG-1 expression by blocking the specific binding of p53 to DNA and inhibiting the transcriptional function of p53 [14]. In cervix carcinoma cells inhibition of PTTG-1 using antisense-ODNs leads to growth inhibition and increased apoptosis [21]. Further, several other studies implicate that PTTG-1 overexpression was associated to chromosomal instability, increased distant metastases, increased neoangiogenesis and invasiveness of the tumor [5,6,22]. These features, induced by PTTG-1 overexpression, would rather cause a more aggressive tumor phenotype. This would be in line with our results showing that increased PTTG-1 expression was significantly correlated to clinical and pathological markers indicating an unfavourable outcome for patients with NSCLC such as extensive lymph node metastases, presence of distant metastases and increased LDH levels. Differences in PTTG-1 expression levels could be translated in a mean survival difference of 5.2 months in the NSCLC patient subgroups which was statistically significant in univariate and multivariate analysis. As a result, PTTG-1 meets the requirements to serve as a biomarker in patients with NSCLCs. In this context a predictive marker like PTTG-1 might be valuable to come to a decision on the still controversially discussed question if a patient with stage I-IIIA NSCLC should be treated with adjuvant chemotherapy or radiation after a histologically confirmed R0-resection of the tumor.

In conclusion, the functions of PTTG-1 are complex and antithetic and might differ from tumor cell to tumor cell. This is examplified at its best by our results showing an association between PTTG-1 expression and overall survival of tumor patients in dependence of lung cancer subtype. We found that a strong expression of PTTG-1 in SCLC was associated with a significant improved outcome for patients. In contrast, a strong expression of PTTG-1 in patients with NSCLC was associated with a significant shorter survival, advanced lymph node metastases and distant metastases. These results underline that a high expression of PTTG-1 plays an important role in biology of lung cancer, which differs in SCLC and NSCLC. Further experiments are needed to understand the different pathways of PTTG-1 in different lung cancer subtypes.

## Authors' contributions

NR has made substantial contributions in the acquisition and analysis of data and carried out the immunohistochemical procedures. HG took part in the immunohistochemical procedures and has been involved in drafting and revising the manuscript. AA and AR carried out the immunohistochemical procedures and the interpretation of these datas. SK performed the statistical analysis. GG, JN, IB, US and RF participated in the coordination of the study and the treatment with patients. HG, RK and RH made substantial contributions to the conception and design of the study. UR has been involved in the coordination of the study, the treatment of the patients and drafting and revising the manuscript. All authors read and approved the final manuscript.
